# A Rapid and Efficient Route to Preparation of Isocyanate Microcapsules

**DOI:** 10.3390/polym9070274

**Published:** 2017-07-09

**Authors:** Yangbao Ma, Yang Jiang, Haiyan Tan, Yanhua Zhang, Jiyou Gu

**Affiliations:** Material Science and Engineering College, Northeast Forestry University, Harbin 150040, China; myb2015dblydx@163.com (Y.M.); jiangyangnefu@163.com (J.Y.); tanzzx168@163.com (T.H.)

**Keywords:** isocyanates, microcapsules, interface aggregation

## Abstract

In this paper, polyaryl polymethylene isocyanates (PAPI) were used as an innovative alternative material to prepare isocyanate microcapsules. PAPI could be used as core materials, which would react with small molecules containing active hydrogen (1,4-butanediol, ethylene glycol, 1,2-diaminoethane etc.). The reaction products of PAPI and active hydrogen would form a shell by interfacial polymerization reaction in an oil-in-water emulsion. Smooth spherical microcapsules of 70 ~ 180 μm in diameter were produced by controlling agitation rate (600 ~ 1200 rpm). High yields (~80%) of a free-flowing powder of PAPI/polyurethane and polyurea capsules were produced with a high isocyanate groups (–NCO) content of 23 wt % as determined by titration analysis. Structural analysis and quality assessments of each batch of microcapsules were performed by using thermogravimetric analysis, Fourier transform infrared spectroscopy and scanning electron microscopy. Preliminary results indicated the microcapsules were stable with only about 20% loss of –NCO detected after one month storage under ambient conditions. This work showed the great potential of novel microencapsulation technique in development of protection of –NCO and in aspects of micro- and nano-structure construction materials.

## 1. Introduction

The fundamental properties of isocyanates (R–NCO) were discovered during the years 1885–1900. However, the practical uses of these compounds were not realized until about forty years later [1,2]. Since isocyanates included in non-formaldehyde class performs well in terms of bonding property, water resistance, aging-resistant performance and fine manufacturability, it has been widely used in the field of coatings and adhesive [3,4,5,6,7,8,9,10]. Also, as results of isocyanates possess the advantages of great reactivity, it has been used in self-healing materials [11,12,13,14,15,16,17,18,19], graphene-based composite materials [20], nanocomposite materials [21] etc. For instance, the hindered urea bonds (HUBs) were synthesized through the reaction of isocyanates with amine, and hydrolysable polymers by quenching of dissociative intermediate-isocyanates [22]. Sasha et al. demonstrated that isocyanates treatment reduced the hydrophilic character of graphene oxide sheets by forming amide and carbomate ester bonds [23]. In addition, Han et al. fabricated highly conductive polymer-composite fibers via a mixture of toluene diisocyanates (TDI) and toluene 2, 4-diisocyanates-terminated polypropylene glycol [21]. Similarly, because of the high activity of isocyanates, there were many drawbacks, such as short service life, inconvenient storage and transportation. In recent years, the main methods to solve the above problems have been the preparation of blocked isocyanates [5,24,25,26,27]. However, due to high unblocking temperature, complicated operation, and releasing the toxic blocking agents in the unblocking process, the applications of blocked isocyanates have been limited [24,28,29,30].

Microcapsules are micro containers (well-defined reaction environments) containing an active liquid or solid ingredient inside as well-defined reaction environments, to protect the material inside from environmental influences [31,32,33]. They were used in various fields just like nanomaterials [34], including agricultural [35], pharmaceuticals [36,37], cosmetic [38], and textile industries [39,40]. Moreover, microcapsules were also used in adhesive, which could control release of additives and adhesives at the right time and the right place and improve the compatibility of certain ingredients to broaden their application fields [34]. A remarkable application of microcapsules in adhesive was protected active substance [41] and used in self-healing materials [19]. In order to protect the isocyanates groups, Yang et al. [42] succeeded in encapsulating the blocked isocyanates into polystyrene and hydroxyl and amine functionalized polymeric nanocapsules. Then, the shell was functionalized with hydrophobic groups to impart more hydrophobicity and enhance the shelf life of the encapsulated –NCO [41]. In addition, due to isocyanates being reactive with water, it was a potential catalyst-free healing agent for self-healing materials when exposed to wet environments [43]. Isocyanate microcapsules, as a type of smart material, were used in structural materials. The first isocyanates (isophorone diisocyanates, IPDI) microcapsules were encapsulated as a healing agent via interfacial polymerization of polyurethane (PU) [44]. Isocyanate microcapsules also demonstrated self-healing activity of isophorone diisocyanate-alkyd varnish coatings (IPDI-AVCs) in protecting steel surfaces [12]. The –NCO functional groups had high reactivity with active hydrogen-containing species (including water) [1,45], therefore there was great value in many fields. Besides, isocyanate microcapsules were prepared by isocyanates, most of which were the TDI, methylene diphenyl diisocyanates (MDI) and IPDI, but the used PAPI was reported relatively few.

In this paper, isocyanate microcapsules were prepared, in which a small amount of isocyanates were lost, but a large number of isocyanates were encapsulated by interfacial polymerization. The prepared isocyanate microcapsules were polyaryl polymethylene isocyanates (PAPI) as core materials and the reaction products, of which PAPI reacted with small molecules containing active hydrogen (1,4-butanediol, ethylene glycol, 1,2-diaminoethane etc.) as shell by via interfacial polymerization reaction in an oil-in-water emulsion. The effects of parameters, including reaction temperature, PAPI concentration, agitation rate and environmental, on the formation of microcapsules were systematically investigated and optimized. Furthermore, structural analysis and quality assessments of each batch of microcapsules were performed using thermogravimetric analysis, Fourier transform infrared spectroscopy and scanning electron microscopy. Moreover, the change of –NCO in the process of preparation and storage environment were particularly concerned in order to provide reference for its application in future.

## 2. Materials and Methods 

### 2.1. Materials

Polyaryl polymethylene isocyanates (PAPI, 44V20) was obtained from Bayer (Leverkusen, Germany). Gum arabic, Sodium dodecyl benzene sulfonate (SDBS), Sodium hydroxide (NaOH) was purchased from Tianjin Kemiou Chemical Reagent Co., Ltd. (Tianjin, China). Di-*n*-butylamine, Ethylenediamine (EDA), 1,4-Butanediol (BDO), Glycerol (GL), Ethylene glycol (EG), *n*-Butyl acetate (BAC) were obtained from Sinopharm Chemical Reagent Co., Ltd. (Beijing, China). Polyethylene glycol 400 (PEG400), Chlorane (HCl), Triethanolamine (TEA) was obtained from Tianjin Fuyu Fine Chemical Co., Ltd. (Tianjin, China) All chemicals in this study were used as received and without further purification unless otherwise specified.

### 2.2. Synthesis of Microcapsules

The isocyanate microcapsule was prepared by interfacial polymerization in an oil and water emulsions or suspensions. At room temperature, 120 g of gum arabic aqueous solution (3%) and SDBS (0.60 g) surfactant were mixed in a 250 mL beaker. The beaker was suspended in a temperature controlled water bath on a programmable hot plate with an external temperature probe. The solution was agitated with a digital mixer (Ren he, Changzhou RH-6BModel, Changzhou, China), driving a two-bladed inclined propeller until the SDBS was dissolved. Then, NaOH (10 wt %) solution was poured into the aqueous phase system to make alkaline system. To prepare microencapsulation solution, 20.00 g PAPI liquid was mixed well with Butyl acetate, and the mixture was then added into the prepared aqueous phase system under agitation and ultrasonifying at 99% amplitude (Kun Shan Ultrasonic Instruments KQ-50DE Model, Kun shan China) to develop a stable emulsion or suspension system. After sonication and agitation, the water bath was heated to preset temperature at a rate of 5 °C/min. At the preset temperature, active hydrogen-containing small molecules as a chain extender were slowly added to the emulsion or suspension to initiate the interfacial polymerization at the oil/water interface. After a certain period of agitation the mixer and hot plate were switched off. Once cooling to ambient temperature, the resultant microcapsules were vacuum filtered and washed with distilled water for several times until the filtrate was neutral. Microcapsules were air-dried at room temperature for 12 h before further analysis.

**Figure 1:**
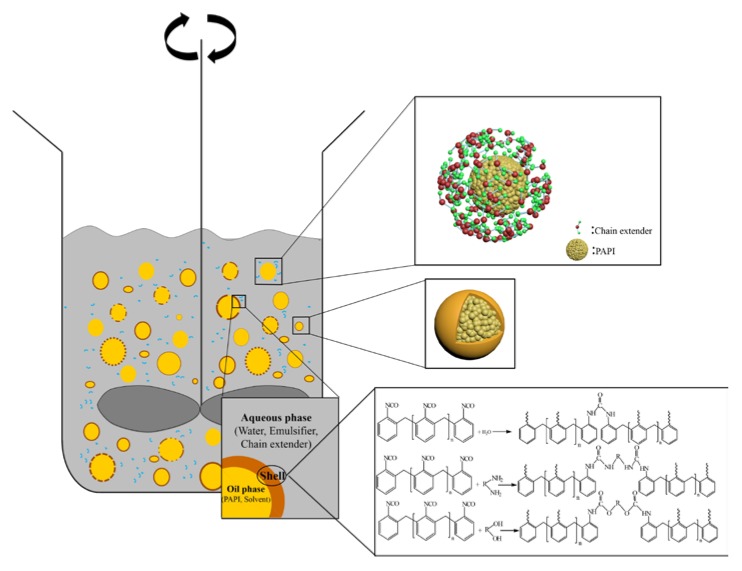
Schematic diagram of capsule shell formation under mechanical agitation and the related chemical reaction.

The PAPI containing –NCO active group and Chain extender containing active hydrogen were dissolved in mutually incompatible BAC and water. Then PAPI was dispersed in aqueous solution to form oil in water emulsions or suspensions, by mechanical agitation and sonication. The polymerization occurred only at the oil-water interface of emulsion droplets, and neither solution BAC nor water of polyurea or polyurethane was formed as microcapsule walls, as schematically shown in Figure 1. Finally, the reaction was terminated by the OM to determine whether or not the capsule was formed. That means the microcapsule wall thickness was controlled by controlling the degree of reaction, so that the microcapsule wall was sufficient to wrap the unreacted PAPI and bear the release force.

### 2.3. Characterization

#### 2.3.1. Yield of Microcapsules

Since synthesis of the microcapsules shell was not a strict stoichiometric reaction, excess chain extender was used to ensure the shell formed, and yield of the synthesis was calculated simply through Equation (1):

(1) $$Yield=\frac{M_{cap}}{M_{PAPI}+M_{CE}}\times 100\%$$

where *M*_cap_ is the mass of collected microcapsules after drying, and *M*_PAPI_, *M*_CE_ are the masses of PAPI and chain extender, respectively. 

#### 2.3.2. Titration of –NCO Content in Samples

The samples were dissolved by acetone with help of a mechanical agitator, and then added in a 250 mL Erlenmeyer flask with a stopper. Di-*n*-butylamine solution (20 mL, 0.1 N, acetone) was added by using a pipet. After swirling for 15 min, bromphenol blue indicator solutions (4–6 drops) were added. Two titrations were performed with hydrochloride acid (0.1 N) to a yellow color end point. Simultaneously, the blank titrations were run including all reagents above but omitting the samples [46]. The NCO content is calculated through Equation (2):

(2) $$NCO,\%=\frac{(V_{O}-V)\times C\times 42.02}{M\times 1000}\times 100$$

where *V*_O_ is the volume (mL) of HCl solution required for the titration of 20 mL di-*n*-butylamine in acetone solution (blank); *V* is the volume (mL) of HCl solution required for the titration of sample; *C* is the molar concentration of HCl solution; *M* is the weight of the sample, g; and 42.02 is the equivalent weight of isocyanates group, g/mol. The titration was repeated three times for each sample and the results were averaged.

#### 2.3.3. Microcapsule Size

Microcapsules diameter and size distribution was examined using an Optical microscopy images with Digital Still Camera (DSC, NETZSCH, SELB, Germany) and image analysis software (Image-Pro Plus 6.0, Media Cybernetics, Rockville, MD, USA). Mean diameter was determined from data sets of at least 200 measurements from OM images.

#### 2.3.4. Morphology of Microcapsules

The surface morphologies and shell thickness of the samples were characterized by using a scanning electron microscope (SEM, FEI QUANTA200, FEI, Hillsboro, OR, America). The SEM was operated at an acceleration voltage of 10 kV. Some samples were cut with razor blades to observe the internal structure of microcapsules. The samples were sputter-coated with gold prior to SEM observation.

#### 2.3.5. Fourier Transform Infrared Spectra Analysis

The chemical structure of the resultant microcapsules was characterized by Fourier transform infrared spectra (FTIR, BRUKER TENSOR II, BRUKER, Karlsruhe, Germany). Meanwhile, Fourier transform infrared spectra analysis also accessorial proof microcapsule structure. Spectra were collected from 400 to 4000 cm^−1^. All the output spectra were the result of the combination of 32 scans for noise reduction. To quantify the core fraction, the core was extracted by stirring microcapsules in *n*-Butyl acetate (BAC). The solvent penetrated the capsule core significantly and swells the shell, releasing the encapsulated core into the solvent. In addition, the microcapsules shells were obtained by filtering the suspension.

#### 2.3.6. Thermogravimetric Analysis

Thermogravimetric analysis (TGA, PERKINELMER STA600, NETZSCH, SELB, Germany) was used to investigate the thermal stability and assisted analysis of the structure of the microcapsules. 2–5 mg of microcapsules were put into an alumina crucible and heated from 30 to 800 °C at a rate of 10 °C/min under nitrogen atmosphere.

## 3. Results

### 3.1. Parametric Study and Optimal Microcapsules Procedure

#### 3.1.1. Determination of Minimum Reaction Time

**Figure 2:**
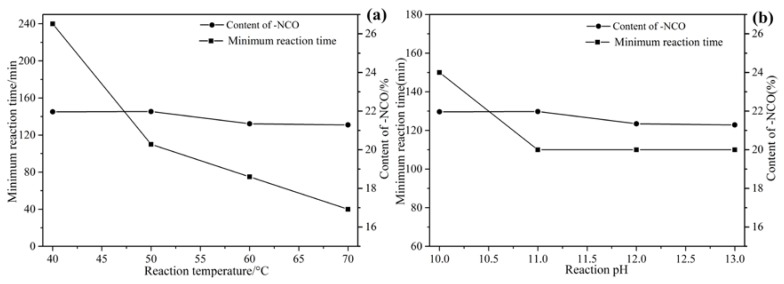
The minimum reaction time and –NCO content with different temperature (**a**) and pH (**b**).

The formation of microcapsules was very fast in the synthesis due to high reactivity of PAPI. However, the minimum reaction time was necessary to know for both time saving and quality control of –NCO content. To determine the minimum reaction time, products were sampled from the emulsions or suspensions at 10 min intervals and observed under microscope until dispersed dry microcapsules could be collected [19]. The required minimum reaction time at different reaction temperature and pH was plotted in Figure 2, and it could be seen that the minimum reaction time reduced steadily from 240 to 40 min when the reaction temperature increased from 40 to 70 °C respectively (Figure 2a). However, the content of –NCO changed little. Although the reaction time was reduced significantly, it was easy clumped at high temperature. Similarly, since alkaline environments promote isocyanates reaction, the minimum reaction time reduced steadily from 150 to 110 min when the reaction pH increased from 9.0 to 13.0 respectively (Figure 2a). Also, the content of –NCO changed little. In summary, the optimum reaction temperature was 50 °C when pH was 11.0.

#### 3.1.2. Influence of Agitation Rate

**Figure 3:**
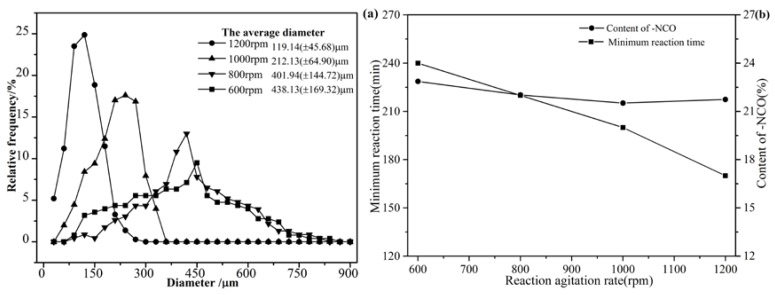
The diameter (**a**), minimum reaction time and –NCO content (**b**) with different agitation rates.

The size of microcapsules was influenced by several factors including the geometry of mixing device, viscosity, blade hydrodynamics, ultrasonic treatment of power and times, and interfacial tension of media, shear agitation rate, temperature, and surfactant effects [1]. The microcapsule size distributions were measured for a range of agitation rates between 600 and 1200 rpm under holding all other factors constant (Figure 3a). With increasing agitation rate, the average diameter of capsules decreased and the size distribution narrowed. This result was also reported by Yang et al. [44] and Brown et al. [46]. At low agitation rate (~600 rpm), interfacial tension was a domination factor, resulting in larger droplets dispersion. Large droplets were broken up into small ones when strong shear forces were experienced under high agitation rate (~1000 rpm) [44]. In addition, faster agitation was more favorable for the homogenization of the emulsion or suspensions. Therefore the diameter distribution of produced microcapsules was more uniform [19]. In summary, the processing conditions for optimal capsule size could be determined according to the application requirements. As shown in Figure 3b, with increasing agitation rate, the minimum reaction time reduced steadily from 240 to 170 min when agitation rate increased from 600 to 1200 rpm respectively. Because the higher rotational speed was, the better dispersion larger reaction contact area had. As a result, thinner shell of the microcapsules was formed (As the enclosed thing was relatively small, the force required was small), and less reaction time would be needed. Since the ratio of shell thickness to capsule diameter was relatively constant, the content of –NCO changed little.

#### 3.1.3. Influence of Solvent

**Figure 4:**
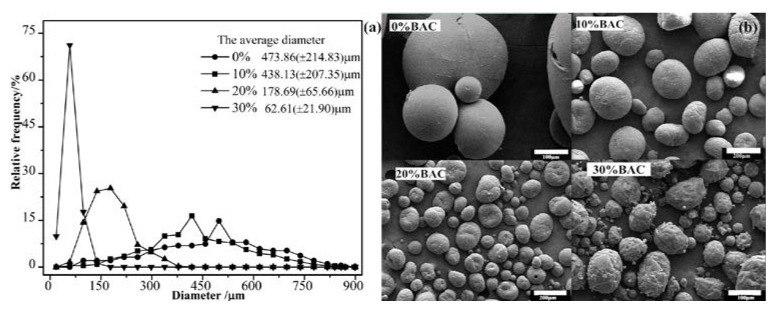
The diameter (**a**) and surface morphology (**b**) of microcapsules obtained at various solvent.

The solvent (Macrogol *n*-Butyl acetate, BAC) of PAPI greatly influenced the diameter and surface morphology of resultant microcapsules. As shown in Figure 4a, microcapsules diameter decreased dramatically when the solvent increased from 0 to 30 wt % (relative to PAPI), which was further proved by SEM images. The solvent influence on capsules formation was interfacial tension of the media. The solvent can reduce the viscosity of PAPI, then the higher solvent concentration formed smaller oil droplets in an oil-in-water emulsion, and as a result, the microcapsules produced via interfacial reaction had smaller diameters. The surface morphology of microcapsules synthesized under various solvents was observed by SEM (Figure 4b). The outer surface of capsules became rough, wrinkle and even cracked with the BAC increased, because of the influence of the contact area between PAPI and chain extender, inhomogeneous reaction kinetics, and solvent evaporation in drying stage,. The addition of solvent had little effect on the internal structure, because the internal isocyanate solution was involved in the reaction, just as the experimental design. To sum up, the amount of BAC can be added according to actual requirements of particle size.

#### 3.1.4. Influence of Chain Extender

**Figure 5:**
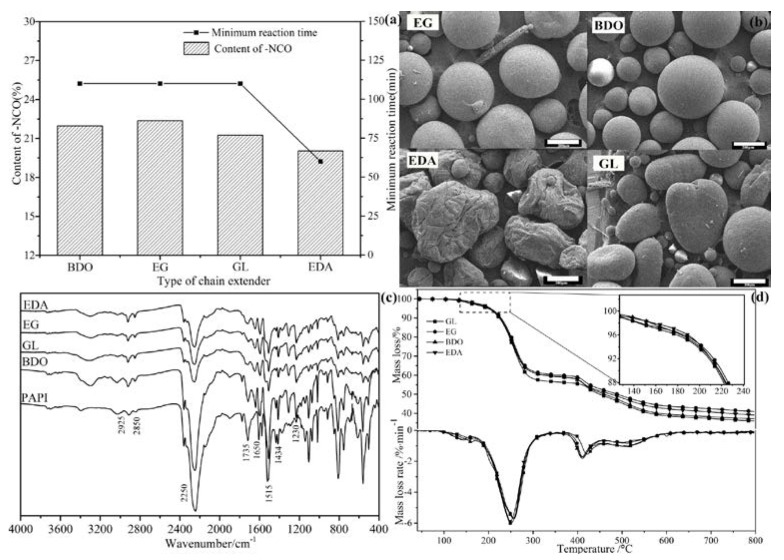
The minimum reaction time with different type of chain extender (**a**), morphology of microcapsules (**b**), Fourier transform infrared (FTIR) spectra (**c**) and thermogravimetric analysis (TGA) mass loss and its derivatives curves (**d**) of microcapsules obtained at various type of chain extender.

The type of chain extender affected the minimum reaction time and morphology. In this paper, alcohols and amine chain extenders were mainly used to prepare microcapsules. In Figure 5a, the content of –NCO of microcapsules prepared by using various chain extenders changed little. The reaction time for prepared microcapsules by EDA required less time than alcohol, but the morphology was not regular and outer surface was not smooth (Figure 5b), result of the higher reactivity with isocyanates than alcohol [2]. The shape of microcapsules prepared by GL was different from microcapsules prepared by EG and BDO, because the chain extenders of GL contain trifunctional, resulting in an inhomogeneous reaction. In addition, interfacial reactions occurred only at the outer surface of the emulsion droplet6, so the type of chain extender had less effect on the internal structure. As the interface reaction products similar or even the same, its chemical structure and thermal stability were also similar, as shown in Figure 5c,d. However, due to the high reactivity of amines, it was added slowly and easy condensed during the preparation process of microcapsules, amine chain extenders were not suitable in this paper. On the morphology, the size of prepared microcapsules by BDO was the most regular. In conclusion, BDO was used as a chain extender in the following studies.

### 3.2. Characterization and Properties of Microcapsules

#### 3.2.1. Yield of Microcapsules

For the microcapsules prepared, it was shown by calculation that the yield was about 80%. It was found that the yields of microcapsules fluctuated slightly, but remained relatively stable under different preparation conditions. It was likely attributable to two reasons. In the first place, the content of –NCO was relatively stable for the prepared microcapsules. It indicated that the degree of reaction has a lot of likeness even sameness. Secondly, during the product collection, a portion of tiny capsules was not collected in the process of filtration and washing, resulting in fluctuant yield. As mentioned above, the yield calculation was a rough estimation. 

#### 3.2.2. Stability of Microcapsules

**Figure 6:**
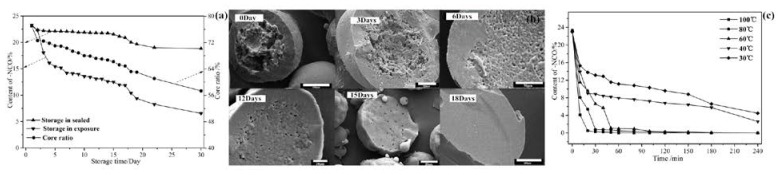
The change of –NCO content and the core ratio of microcapsules for different time (**a**). Scanning electron microscope (SEM) images of microcapsules at different storage time (**b**). The change of the –NCO content of microcapsules was immersed for different time and temperature (**c**).

To investigate the storage stability of microcapsules in environment, the capsules were analyzed by after they were closed and exposed in environment of room temperature 25 °C, humidity about 65% for a period of time. As revealed in Figure 6a, the content of –NCO decreased from 23% to 18%, while at the same time, the core ratio decreased from 77% to 57% when the samples were sealed, while the content of –NCO decreased from 23% to 6% when the samples were stored within 30 days. It indicated that the isocyanate microcapsules have good stability. The decline of the –NCO content and core ratio can be explained as follows: moisture diffused across the microcapsule shell and reacted with the –NCO of encapsulated PAPI or there was a subsequent reaction with active hydrogens of carbamates and substituted urea, etc., which was further proved by the SEM images of the change of the core morphology of the same batch of microcapsules at different storage time. As shown in Figure 6b, the core materials were solidified and eventually formed solid particles.

Moreover, to search the use of stability of microcapsules in a wet environment, the microcapsules were analyzed by chemical titration after they were immersed in different temperatures water for a period of time, as shown in Figure 6c. The content of –NCO reduced with the immersion time and temperature, and the temperature was raised and the faster it fallen. The reason was described above.

#### 3.2.3. Morphology of Microcapsules

**Figure 7:**
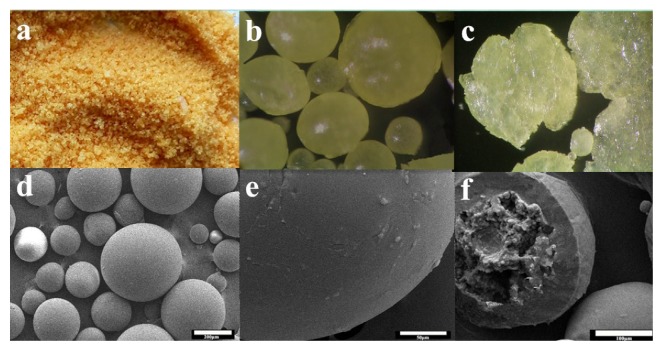
Morphology of microcapsules: (**a**) Digital camera image(Canon, Tokyo, Japan); (**b**) OM image( 100×); (**c**) OM image of flattened microcapsules(100×); (**d**) SEM micrograph with spherical shaped microcapsules; (**e**) zoomed in image showing smooth outer surface; and (**f**) micro structure morphology.

The surface and microstructure morphology of microcapsules were shown in Figure 7. The capsule shape was nearly spherical and they were individually distributed without excessive conglomeration among each other (Figure 7a). The microcapsules were optically transparent (Figure 7b) and could be easily squashed (Figure 7c) due to the low mechanical strength. It also indicated that the microcapsules shell was soft and deformable, and then imparted flexibility and tightness. As a result, the PAPI liquid was effectively encapsulated. The surface, shell and microstructure morphology of microcapsules were observed by using SEM (Figure 7d–f). It could observe the difference between smooth outer and inner rough surface (Figure 7e,f). The boundary was not distinct (Figure 7f) resulting from the interaction of inhomogeneous reaction kinetics [47], or the degree of reaction of PAPI emulsion droplets with the chain extender was different. Because the reactions outside of emulsion droplets were complex, while the inside of the emulsion droplets without reactions was regard as microcapsules core during the formation of encapsulated process.

#### 3.2.4. Chemical Characterization by FTIR

**Figure 8:**
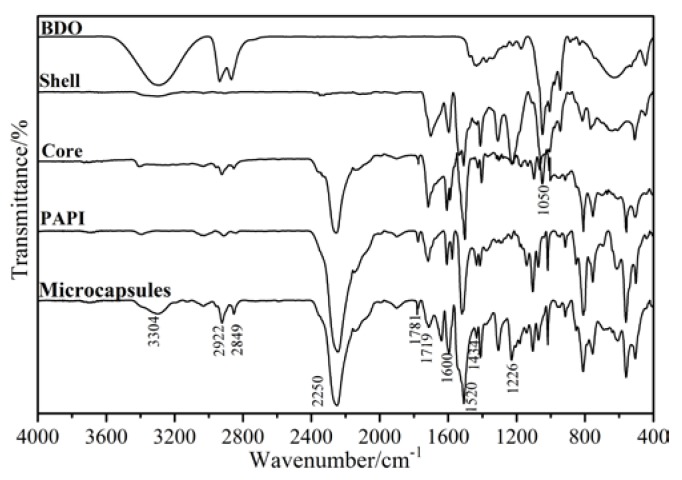
FTIR spectra of microcapsules, capsules shell, capsule core, 1,4-Butanediol (BDO) and polyaryl polymethylene isocyanates (PAPI).

The chemical structure of resultant microcapsules was characterized by FTIR. For comparison, complete capsules together with pure grades of PAPI, 1, 4-Butanediol (BDO), shell and core materials were tested, as shown in Figure 8. The nearly identical spectrum curves of PAPI and core material indicated that PAPI was successfully encapsulated. From the spectrum of shell, the characteristic signal at 2250.6 cm^−1^ (–NCO stretch) was not observed, indicating the PAPI were reflected with BDO to form polyurethane or polyurea shell. At the same time, the microcapsules and its shell were monitored by using infrared absorption peaks, as assigned to the urea group (–NH–COO–) at 1226 cm^−1^, and 1434 cm^−1^ (–C–N). Furthermore, the microcapsules, including the core material, appeared in the large the –N=C=O stretch vibration peak at 2270 cm^−1^ of the spectrum of complete capsules. Thus, it was logical to confirm again that PAPI was successfully encapsulated, and all microcapsules contained PAPI, which was the core of microcapsules.

#### 3.2.5. Thermogravimetric Analysis

**Figure 9:**
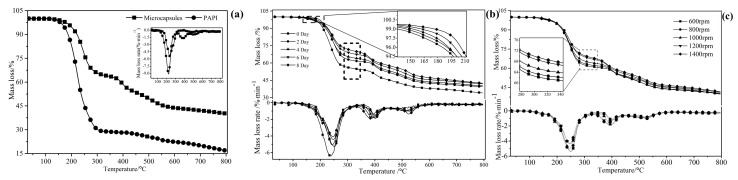
TGA mass loss and its derivatives curves of PAPI and synthesized microcapsules (**a**), changed in different storage time at room temperature (**b**) and by synthesized at various agitation rate (**c**).

The thermal properties of filled microcapsules and pure PAPI were characterized by TGA. The resultant curves of mass loss for material were presented in Figure 9a. The pure PAPI decomposes mostly (loses about 70%) at 223 °C. However, there were also present the same peaks at the same temperature for the microcapsule, it was alluded all microcapsules contain PAPI as core. With rising of temperature, the microcapsules were degraded gradually and its degradation rate was the same as PAPI (front part). Combined with the core rate, the degradation of PAPI and microcapsules were the same by calculation (front part). It well confirmed microcapsules contained PAPI. 

In addition, the thermal properties of the same batch of microcapsules were characterized by TGA at different times, as shown in Figure 9b. The results pointed that, as time went on, the initial decomposition temperature gradually increased, and the mass loss was also reduced at temperature of PAPI decomposed. It meant that core materials were gradually reduced due to continued chemical reactions as the above explanation of stability. Meanwhile, this change regularity was consistent with the core rate above. Furthermore, the thermal properties of microcapsules made at various agitation rate were also characterized by TGA after prepared for a period of time (about 3 days), as shown in Figure 9c. The results showed that the mass loss decreased with the increasing rotational agitation rate, resulting in the small particles have larger contact area in continuous reaction.

## 4. Conclusions

In this study, isocyanate microcapsules were successfully prepared by interfacial polymerization in an oil-in-water emulsion or suspension. Partial core material PAPI reacts with small-molecule active hydrogen to produce polyurethane and polyurea that act as the shell of microcapsules. The whole synthesis process didn’t need adding any material just for a shell material, provided a new, time-saving and energy-efficient strategy. The resultant microcapsules were in a satisfactory spherical shape with high yield, and the particle size could be controlled by changing rotational speed and amount of solvent. In addition, the optimum preparation method was achieved by changing parameters including reaction temperature (40 ~ 70 °C), solvent addition (0 ~ 30%), agitation rate (600 ~ 1200 rpm), environmental pH (9 ~ 14), etc. The prepared isocyanate microcapsules had obvious core—shell structure and good stability by interfacial polymerization. In summary, this study showed that the prepared isocyanate microcapsules successfully encapsulated liquid PAPI in polyester and polyurea. This work showed great potential in self-repairing materials and structural smart materials, etc.
